# Characterizing storm-induced coastal change hazards along the United States West Coast

**DOI:** 10.1038/s41597-022-01313-6

**Published:** 2022-05-23

**Authors:** James B. Shope, Li H. Erikson, Patrick L. Barnard, Curt D. Storlazzi, Katherine Serafin, Kara Doran, Hilary Stockdon, Borja Reguero, Fernando Mendez, Sonia Castanedo, Alba Cid, Laura Cagigal, Peter Ruggiero

**Affiliations:** 1 University of California at Santa Cruz, Institute of Marine Sciences, Santa Cruz, California USA; 2 U.S. Geological Survey, Pacific Coastal and Marine Science Center, Santa Cruz, California USA; 3 University of Florida, Department of Geography, Gainesville, Florida USA; 4 U.S. Geological Survey, St. Petersburg Coastal and Marine Sciences Center, St. Petersburg, Florida USA; 5 Universidad de Cantabria, Department of Sciences and Techniques in Water and Environment, Santander, Spain; 6 Oregon State University, College of Earth, Ocean, and Atmospheric Sciences, Corvallis, Oregon USA

**Keywords:** Natural hazards, Physical oceanography

## Abstract

Traditional methods to assess the probability of storm-induced erosion and flooding from extreme water levels have limited use along the U.S. West Coast where swell dominates erosion and storm surge is limited. This effort presents methodology to assess the probability of erosion and flooding for the U.S. West Coast from extreme total water levels (TWLs), but the approach is applicable to coastal settings worldwide. TWLs were derived from 61 years of wave and water level data at shore-perpendicular transects every 100-m along open coast shorelines. At each location, wave data from the Global Ocean Waves model were downscaled to the nearshore and used to empirically calculate wave run-up. Tides were simulated using the Oregon State University’s tidal data inversion model and non-tidal residuals were calculated from sea-surface temperature and pressure anomalies. Wave run-up was combined with still water levels to generate hourly TWL estimates and extreme TWLs for multiple return periods. Extremes were compared to onshore morphology to determine erosion hazards and define the probability of collision, overwash, and inundation.

## Abstract (summary)

**Table:** 

Measurement(s)	Total Water Levels • Coastal Erosion Probabilities
Technology Type(s)	Numerical Wave models • SWAN, Matlab
Factor Type(s)	Wave Heights • Wave Directions • Wave Periods • Tides • Morphology
Sample Characteristic - Environment	shoreline
Sample Characteristic - Location	U.S. West Coast

## Background & Summary

The U.S. Geological Survey’s (USGS) National Assessment of Storm-Induced Coastal Change Hazards developed methods to identify coastal change hazards affecting the U.S. East and Gulf Coasts (East Coast, hereafter)^1,2^. Some of this methodology is transferable to the U.S. West Coast (West Coast, hereafter), but many of the physical drivers of flooding and erosion differ due to geologic setting (for example, narrow versus wide continental shelves, coasts with high-relief cliffs versus low-relief passive margin dune systems) and variations in storm generation and types (extratropical cyclones versus tropical cyclones). Much of the West Coast lacks consistent, regional scale, event-driven coastal change and hazard assessment data.

To fill this gap, this study developed a coastal hazards assessment framework suitable for the West Coast to provide consistent, event-driven coastal flooding and erosion hazard assessments at a resolution of 100 meters along the open coast. The active tectonic processes of the West Coast create an extremely diverse coastline composed of partially lithified sea cliffs and bluffs, extensive dune fields, sandy beaches, and resistant headlands that break the shoreline into a set of weakly connected littoral cells^3^. Sea cliffs and bluffs compose the majority of the coastline, but sandy beaches are also common, with coastal dunes comprising approximately 45% of the Oregon and Washington outer coasts^4^. An alongshore resolution of 100-m was selected to adequately capture the variability of the West Coast on the municipality-to-regional scale and match the resolution of prior USGS CoSMoS coastal flood modeling efforts in Southern California for consistency between USGS products^5–7^. Additionally, we selected this alongshore resolution to provide an ambitious level of spatial coverage over the more than 2000 km of coastline while still being practical for data storage capacity, model processing time, and regional calibrations.

Hydrodynamic forcing varies between the East and West Coasts, with typical tides and waves approximately two times higher on the West Coast compared to the East Coast. The West Coast has a strongly seasonal wave climate, with waves significantly elevated during the winter months due to extratropical cyclones in the eastern North Pacific^8^. Along the Pacific Northwest (Washington through Northern California), extreme significant wave heights reach or surpass 10 m at least once a year^9,10^, whereas southern California annual high wave events are closer to 6 m^9^. Along the East Coast, the historical mean and 95^th^ percentile significant wave heights range from 1.5–2 time lower on average^11^. The average tidal range along the open coast ranges from, 1.13 m in Southern California to 1.93 m in Northern Washington state. The East Coast has a much larger tidal range of ~4 m in Maine decreasing south to 0.4 m in the Florida Keys (https://tidesandcurrents.noaa.gov/). Storm surge and coastal flooding on the East Coast are dominated by storms such as hurricanes and Nor’easters, the equivalent of which do not typically make landfall on the West Coast. Combined with its narrow continental shelf, storm surge is relatively modest on the West Coast (on the order of 1 m maximum) compared to the East Coast (where storm surge may exceed 3 m during a hurricane). Its seasonal water level variations are largely tied to upwelling and downwelling-favorable winds and are coupled to water temperature variations that can also affect localized sea level anomalies^12^.

Finally, coastal hazards on the West Coast are greatly influenced by strong, interannual oceanic and atmospheric variability every five to seven years associated with the warm phase of the El Niño-Southern Oscillation (ENSO), when a band of warm ocean water develops in the east and/or east-central equatorial Pacific^9,13–17^. During these El Niño events, the West Coast experiences ~30% larger wave energy than a typical winter^17,18^ and elevated sea level anomalies on the order of 0.2–0.3 m for months at a time^9,17^. These higher-than-average sea levels are a result of the offshore water being abnormally warm, geostrophic effects of stronger northward flowing currents, and the passage of coastal-trapped waves^9,19^.

The geologic and hydrodynamic differences between coastlines of the East and West Coasts introduce a new set of challenges to the USGS’s National Assessment of Storm-Induced Coastal Change efforts, particularly in characterizing coastal morphology, defining storm “scenarios,” and determining representative extreme water levels. This project addresses these challenges by presenting a comprehensive methodology to assess West Coast storm-induced coastal change hazards, and fills in the gap to complete the conterminous United States-scale hazard assessments, joining the East and Gulf Coast products^1,2^.

This methodology accompanies the release of a storm-induced coastal change hazard assessment, based on the USGS’s storm impact scale^20^, that determines the probability of erosion of coastal features such as dunes, overwash of these features, and inundation (pCOI) of backing topography on open, exposed shorelines of the United States West Coast. These data include mean high-water estimates, dune/barrier toe and crest elevations at 100-m alongshore increments. Additionally, these data include projected total water levels (TWLs) and dynamic water levels (DWLs) for the 1-, 2-, 5-, 10-, 20-, 25-, 50-, 100-, 250-, and 500-year return period events along with the probability of each storm impact scale regime occurring. DWL here is defined as the combined water surface elevation due to the still water level (described later in this document), wave setup, and infragravity wave motions. TWLs are calculated as the total wave run-up elevation above DWL. Water level calculations are discussed in more detail in the *Run-up Calculations by Shoreline Type* section. Finally, a days-per-year projection of each regime is included for each location.

## Methods

This effort estimates the probability of coastal change associated with extreme total water levels (TWLs) over a range of return periods, including the 1-, 2-, 5-, 10-, 20-, 25-, 50-, 250-, 100-and 500-year events, using the pCOI scale. These probabilities are further refined into a days-per-year analysis of TWL impacts. Due to the nature of storms on the West Coast, a return period approach is more appropriate for evaluating extreme TWLs rather than focusing on singular events, such as hurricanes on the East Coast. Focusing on return-period events allows the evaluation of all possible types of storms, both of greater and lesser extremes (such as TWLs from annual winter storms). Additionally, a return period approach was selected (as opposed to investigating joint wave-water level conditions) because return periods are a metric familiar to coastal managers and are analogous to coastal flooding products produced by the United States’ Federal Emergency Management Agency (FEMA). This process is described in detail below.

**Fig. 1:**
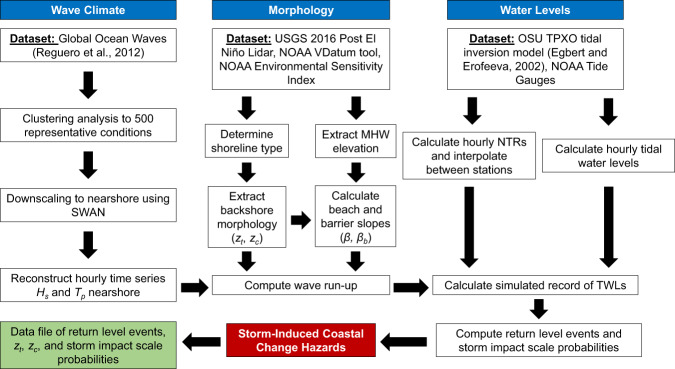
Flow chart explaining the methodology employed in this study. The blue boxes indicate the individual components needed for the study, the red box indicates the final calculated product, and the green box indicates data available for download. The abbreviation NTRs represent non-tidal residuals described in the Extreme Total Water Levels section.

To develop a database of extreme TWLs and pCOI estimates, we first generated regionally consistent estimates of coastal water levels, waves, and morphologic characteristics. To do this, we cast consistently spaced transects along all open shorelines of the West Coast. At each transect we characterized the local shoreline type and extracted morphologic features such as dune crest/toe and beach slope from the high resolution USGS/ National Oceanic and Atmospheric Administration (NOAA) 2016 Post El Niño LiDAR elevation dataset. Wave run-up and water level components were numerically and statistically modeled at hourly time steps to yield a combined time series of TWLs at the shoreline along each transect. Return period events were calculated by extreme value analysis for each TWL timeseries and subsequently compared to the onshore morphology to determine the pCOI regimes. These steps and associated datasets are briefly described in Fig. 1 and in more detail in subsequent sections.

### Morphology

#### Cross-Shore Transects and Shoreline Type

Shore-perpendicular transects were generated at a 100-m alongshore resolution for open coast locations stretching from the strait of Juan de Fuca, Wash., to the US-Mexico Border in San Diego, Calif., and were designated as Major Transects. These transects extended offshore to the 15 m water depth contour or to a maximum distance of 3 km offshore if the 15 m contour was not intersected. The transects were extended up to 300 m onshore to capture relevant morphology. This distance was determined by testing for extremely wide beaches where a dune or backing features was very far from the shoreline. Transect elevation profiles started at the first instance where the local mean high water (MHW) elevation, as extracted from the NOAA VDatum tool^21^, intersected the topography and progressed landward. The transect did not extend landward past any elevation lower than MHW, such as locations with a small backing bay or riverine system. For the purposes of this study, the MHW location represented the shoreline as the LiDAR dataset was limited to the subaerial topography at the time of capture, with the water surface often obscuring the morphology lower than MHW. The transects for California were derived from pre-existing Monitoring and Prediction profiles established by the Scripps Coastal Data Information Program^22,23^, which cover California’s coast at an approximate alongshore spacing of 200 m. Roughly half of the transects were co-located with the Monitoring and Prediction profiles and additional transects were cast in between to increase the alongshore resolution to 100 m. In Oregon and Washington, transects were generated perpendicular to the general shoreline angle derived from the NOAA West Coast Continually Updated Shoreline Product shapefile^24^ every 100 m alongshore to mirror spacing and orientation considerations in the Monitoring and Prediction transects. These profiles were subsequently adjusted manually in ArcGIS to ensure that each was perpendicular to the shoreline and account for shoreline crenulation. For example, transects that were not oriented towards open water, such as in small embayments, were removed. A series of Minor Transects were then cast between the 100-m spaced Major Transects at approximately 10-m alongshore resolution for the whole of the West Coast.

To accurately calculate wave run-up and, ultimately, interpret extreme TWL impacts, it was necessary to determine the shoreline type (for example, sandy beach, sea cliffs, or engineered structures) at each major transect. Shoreline types were identified by a combination of visual identification using GIS software, coastal armoring geospatial data^25^, and NOAA’s environmental sensitivity index (ESI) geospatial data^26–30^. While the ESI data were originally conceived to determine shoreline sensitivity to oil spills, this index of shoreline physical parameters (Table 1) is useful for determining shoreline types at large scales.

**Table 1:** ESI categories found within the study domain along the U.S. West Coast and their associated physical descriptions.

ESI	Shoreline Type
1A	Exposed Rocky Shores
1B	Exposed, Solid Man-made Structures
2A	Exposed Wave-cut Platforms in Bedrock
3A	Fin-to Medium-grained Sand Beaches
3B	Scarps and Steep Slopes in Sand
4	Coarse-grained Sand Beaches
5	Mixed Sand and Gravel Beaches
6A	Gravel Beaches
6B	Riprap
6D	Boulder Rubble
7	Exposed Tidal Flats
8A	Sheltered Rocky Shores
8B	Sheltered, Solid Man-made Structures
8C	Sheltered Riprap
8F	Vegetated, Steeply Sloping Bluffs
9A	Sheltered Tidal Flats
9B	Vegetated Low Riverine Banks
9C	Hypersaline Flats
10A	Salt- and Brackish-water Marshes
10B	Freshwater Marshes
10C	Swamps
10D	Scrub-Shrub Wetlands

#### LiDAR-Derived shoreline morphology analysis

**Fig. 2:**
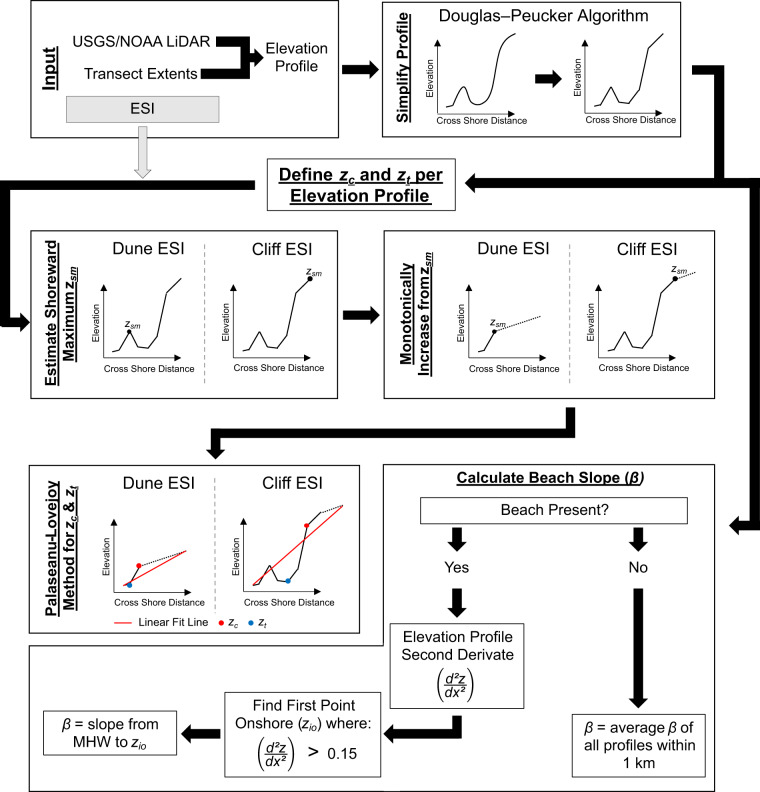
Flow chart detailing the LiDAR-derives shoreline morphology analysis from input LiDAR dataset, profiles locations, and Environmental Sensitivity Index (ESI) category. Note there are two separate calculation branches: one to evaluate dune/cliff crest (*z*_*c*_) and toe (*z*_*t*_) and another to calculate beach slope (*β*) for a given profile. *z*_*sm*_ represents the intermediate calculation value of the most shoreward maximum along the simplified profile, *d*^*2*^*z/dx*^*2*^ is the second derivative of the simplified elevation profile, and *z*_*io*_ is the first onshore point where the second derivative is >0.15 to define the upper bound for *β* calculation (as determined by testing).

**Fig. 3:**
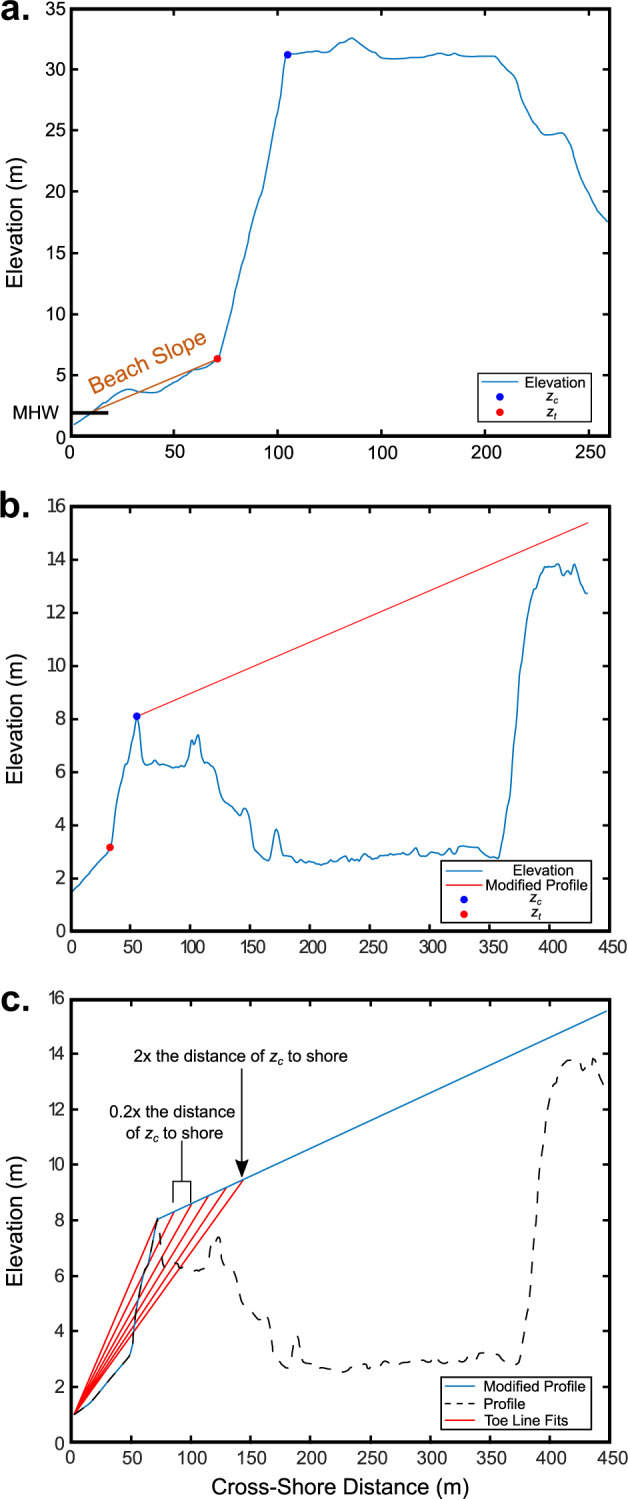
Example elevation profiles in Santa Cruz County, Calif. of a cliff-backed beach (**a**) and a dune (**b** and **c**), with identified cliff and dune crest locations (*z*_*c*_) and toes (*z*_*t*_) locations relative to NAVD88 highlighted in blue and red, respectively. (**a**) Example beach slope (*β*) calculation for use with the Stockdon and others (2006) run-up formulation extending from the MHW location along the profile to *z*_*t*_, representing the cliff toe. (**b**) Example dune elevation with a modified elevation profile is shown in red, creating a continuous sloped profile onshore of the dune crest. (**c**) Dune elevation profile simplification and application of the Palaseanu-Lovejoy and others (2016) iterative adaptation to determine *z*_*t*_ location. The dashed black line represents the original cross-shore morphology, the blue line represents the modified morphology to highlight the dune, and the red lines represent fit lines to iteratively identify *z*_*t*_.

The West Coast offers a wide range of shoreline morphologies that are not common along the East Coast, such as plunging cliffs and dune/beach-fronted cliffs. Previous methods for extracting relevant morphologies along the East Coast could not be applied in this setting; therefore, new methods to extract relevant features were developed Fig. 2. Topographic profiles were extracted at each Major and Minor Transect from the USGS/NOAA 2016 post El Niño LiDAR^31^ at a 1-m horizontal resolution. For each profile, morphological features were extracted such as the toe of a dune/cliff/protection structure (*z*_*t*_) or the crest of a dune/cliff/protection structure (*z*_*c*_), as shown in Fig. 3a.

The elevation profile of each transect was first simplified using a Douglas–Peucker algorithm^32^. The algorithm takes a curve composed of multiple points and produces a similar curve consisting of a subset of the original points effectively removing small-scale variations while maintaining the larger structure of the curve. The algorithm simplifies a curve to a user defined degree, which was made variable within this study based on shoreline type. For example, a sandy beach in southern California was not simplified as much as a cliff-backed beach in northern California as too much detail (such as very low-lying dunes or berms along the southern California beaches) would be lost. This process removed small-scale variations in each profile, accentuating desired features, such as dune/cliff/structure crests and toes, to facilitate automatic identification in cross section for use in run-up and pCOI calculations. From this simplified elevation profile, the most shoreward local maximum (*z*_*sm*_) was extracted. Along sandy beach transects, *z*_*sm*_ provided a first approximation of the dune crest, if present. Along cliff profiles, *z*_*sm*_ was often shoreward of the cliff crest and the estimation needed further processing.

In cliff/bluff/engineered environments, *z*_*c*_ was difficult to reliably extract from the simplified monotonically increasing elevation profiles as *z*_*sm*_ did not always align with the precise location of *z*_*c*_. The elevation profile simplification approach was therefore further modified by adapting the methodology of Palaseanu-Lovejoy and others^33^, originally developed for automatically delineating sea cliffs. This approach extracts a potential *z*_*c*_ by detrending the elevation profile and extracting the greatest value. Detrending is done by fitting a line between the first and last point of a profile, subtracting that line from the transect elevations, and extracting the greatest value. The approach works well for cliffs, bluffs, and any feature with a large change in elevation over a small distance, like the one shown in Fig. 3a.

The Palaseanu-Lovejoy and others^33^ approach was also used to identify *z*_*c*_ in non-cliff environments (such as a dune). The methodology was further modified to more accurately identify the *z*_*c*_ after determining *z*_*sm*_. If the elevation change of a feature (like a low elevation dune) is much smaller compared to the total length of the elevation profile (for example, a mild slope), it becomes difficult to identify key features. To ensure that *z*_*c*_ of the relevant feature was reliably identified, *z*_*sm*_ (or if non-existent the maximum elevation of the profile) was used to represent the new local maximum elevation and everything onshore was assigned a gently sloping elevation increase to mimic a monotonically increasing curve. This modification to the elevation profile ensures that the feature of interest now generates the greatest slope and elevation change along the profile after detrending. If the example dune were to be backed by a cliff, the un-adapted methodology would result in *z*_*c*_ of the cliff being the identified feature, ignoring the dune. Often selecting *z*_*sm*_ would be enough to capture the dune crest, but in the case of very complex dune systems, using the adapted Palaseanu-Lovejoy and others^33^ method proved to reliably select the primary fronting dune. Using the above methods, *z*_*c*_ of the dune fronting a cliff is selected (Fig. 3b).

The *z*_*t*_ for dunes, cliffs, and engineered structures (such as riprap) was also cataloged using an adapted version of the Palaseanu-Lovejoy and others^33^ approach. Where there was a monotonically increasing elevation profile with a cliff, the *z*_*t*_ was selected as the lowest value after subtracting the linear interpolation line between the first and last points of the profile. Along profiles with smaller elevation changes, this was adapted by curtailing the cross-shore distance of the profile to *z*_*c*_ and again replacing the remaining distance of the elevation profile with a gently increasing slope, mimicking the monotonically increasing profile shape (shown along a cliff-fronting dune in Fig. 3c). Additionally, the length of the elevation profile was limited to twice as far from the shoreline as *z*_*c*_. If the profile length was much longer than the position of *z*_*c*_, the change in elevation at the feature becomes less pronounced and *z*_*t*_ selection less reliable. Next, a line connecting the first point in the profile to the location of 2 times the distance of the *z*_*c*_ onshore was cast, and if no *z*_*t*_ value was found seaward of the maximum, the endpoint of this line was moved seaward by 0.2 times the distance onshore of *z*_*c*_ (Fig. 3c). This process was repeated until a toe greater than 2.5 m in elevation and seaward of the *z*_*c*_ was selected or until the endpoint of the line became *z*_*c*_. The toe threshold was determined through testing to best represent the *z*_*t*_ in run-up equations and also ensure that *z*_*t*_ was greater in elevation than MHW, determined to be the minimum elevation that could be used to calculate beach slope. The selected minimum of this iterative approach then yielded the feature *z*_*t*_. If the feature was a revetment, for example, then *z*_*t*_ would be considered where the beach sand meets the riprap.

There were specific alterations to this approach with different shoreline features. Along shorelines with detached/freestanding seawalls, whose representation in LiDAR data was often muted, the location of the seawall was identified using GIS data, either derived from previous studies^34,35^ or from satellite imagery. In these cases, z_*c*_ was defined as the location of the seawall crest, as information concerning overtopping of the seawall was deemed most important when present. Even if the seawall was not the most topographically prominent feature, it was selected as the location of greatest importance. From there, the *z*_*t*_ was identified as the closest seaward concave-up inflection point. If there was no identified point, then *z*_*t*_ was selected to be 3 m seaward of the seawall location. If the shoreline was identified as one containing exposed rocky platforms, the *z*_*t*_ was set to be the shoreward extent of the platform. And in the run-up calculation, the platform was treated as a berm. Along plunging cliff shorelines, *z*_*t*_ was set to be mean sea level (MSL), and the location of *z*_*t*_ was estimated from the slope of the cliff.

Beach slope (*β*) was calculated as the slope from the MHW shoreline to the first inflection point shoreward of the MHW location (Fig. 3a) with a second derivative value greater than 0.15 (as determined by sensitivity testing) for all Major and Minor transects. This point generally coincided with *z*_*t*_, but in some cases, such as small foredunes or manicured beaches in Southern California, this inflection point represents the backshore transition point from sandy slope to the backing environment (urban, vegetated, or small dunes). Additionally, some larger dune systems’ *z*_*t*_ elevations were high as determined by the automatic extraction of the elevation profile from the LiDAR. While it is important to know the elevation of those dune toes for analyses, using anomalously high elevations to calculate *β* was problematic as it led to an overestimation of *β*. Limiting the calculation of *β* to a seaward inflection point if the extracted *z*_*t*_ was anomalously high yielded more realistic beach slopes for run-up calculations.

Given that the USGS/NOAA West Coast Post El Niño LiDAR did not extend below the water line at the time of capture, a measurement of MHW to a prominent inflection point along the elevation profile was determined to be the best approximation for *β* for use with run-up formulations described below. Stockdon and others^36^ defined *β* for use within the run-up equation for dissipative beaches as the average slope between ± 2 standard deviations of wave setup during a measured period. Often, during low tides, this method of calculating *β* could not be applied, as elevations lower than MHW were not consistently represented in the LiDAR dataset. Approximating *β* from MHW to a prominent inflection point also represent the maximum onshore slopes that the largest run-up conditions would act over. *β* was determined at each Major Transect using the average of the Minor Transect *β* within 100 m of and including the Major Transect. Along transects where there was no identifiable beach, the *β* for use with run-up equations was calculated as an average regional *β* of all Major and Minor Transects within 500 m up-shore and down-shore of the location.

### Extreme total water levels (TWLs)

Extreme TWLs are used to represent the hydrodynamic forcing during large events, including the potential for enhanced erosion, greater onshore wave attack, and inundation of shoreline-backing environments. It is used within this study to approximate these effects without computationally expensive flood modeling. Along the West Coast, extreme water levels are not always tied to local storms, such as hurricanes on the East Coast. Large waves with the potential for greater flooding and erosion are often generated from extratropical cyclones far afield. Therefore, estimations of return level extremes at each transect were created from the time series of TWLs. This approach is useful along the West Coast when a singular driving factor (such as a hurricane) is absent.

Time series of TWLs were determined by linear superposition of four sea-level components following Serafin and others^37,38^, detailed as:

1 $$TWL=MSL+{\eta }_{A}+{\eta }_{NTR}+{R}_{2{\rm{ \% }}}$$

MSL is mean sea level relative to the North American Vertical Datum of 1988 (NAVD88). Within this study, MSL was extracted from the NOAA VDatum tool and used as a baseline from which all of the other TWL components varied. *η*_*A*_ is the water level anomaly due to astronomic tides, and *η*_*NTR*_ is the water level anomaly due to non-tidal residuals (i.e., any elevation changes to the measured water level not due to the tide, including both seasonal effects and storm surge). An hourly, 61-year time series of *η*_*A*_ was deterministically modeled every 1 km alongshore and *η*_*NTR*_ was statistically modeled at a number of NOAA tide gauges along the West Coast. Collectively, the combination of water level components without wave action represents the still water level (SWL), which is referenced throughout the remainder of this document and defined as: SWL = MSL + *η*_*A*_ + *η*_*NTR*_. *R*_*2%*_ is the 2% exceedance wave run-up, which includes the effects of wave swash combined with the water surface elevation setup from wave radiation stress. R_2%_ is calculated relative to the existing SWL conditions incorporating calculated infragravity and incident wave swash (described in the section *Run-up Calculations by Shoreline Type*). The TWL elevation is output relative NAVD88. The input wave conditions were numerically downscaled to the nearshore at each transect from the Global Ocean Waves (GOW) model^11^ to achieve a 61-year time series of hourly wave conditions to calculate *R*_*2%*_. *R*_*2%*_ is also modulated by shoreline slope and the reflectivity of the onshore morphology, which was determined in the LiDAR derived morphology analysis. Each of these components and how they were calculated are discussed in detail below. Once these components were combined, TWL return periods were calculated from extreme value analyses of hourly TWL time series. The TWL associated with the selected return periods (1-, 2-, 5-, 10-, 20-, 25-, 50-, 100-, 250-, and 500-years) were used to compute storm impact scale probabilities for each transect along the West Coast.

#### Water level inputs

The first component of Eq. 1 to calculate TWLs is to determine the nearshore SWL at each location. First, MSL relative to NAVD88 at each transect was estimated using NOAA’s VDatum tool^21^. η_A_ was calculated at a 1 km alongshore resolution using Oregon State University’s global ocean tide model, Topex Poseidon Crossover Solution version 9.1^39^. Tidal outputs did not significantly vary at the 1-km scale; therefore, tide time series for each profile was assigned as the nearest 1-km spaced output point. The tidal data were modeled at hourly increments from 1948–2008 to coincide with the time period of the GOW model^11^.

The last component of the SWL data was η_NTR_, calculated as the sum of the monthly mean sea level (MMSL) and storm surge (SS) anomalies. Time series of MMSL and SS were calculated by relating these water level anomalies to principal components (PCs) of sea-level pressure (SLP) and sea surface temperature (SST) fields following the methods of Anderson and others^40^. These values were calculated at NOAA tide gauge stations (Table 2) and linearly interpolated between tide stations to coincide with Major Transect locations.

**Table 2:** Name and location of NOAA tide gauge stations for the U.S. West Coast.

Station	Latitude (°N)	Longitude (°E)
San Diego, CA	32.71	−117.17
La Jolla, CA	32.87	−117.26
Los Angeles, CA	33.72	−118.27
Santa Monica, CA	34.01	−118.50
Santa Barbara, CA	34.40	−119.69
Oil Platform Harvest, CA	34.47	−120.68
Port San Luis, CA	35.17	−120.75
Monterey, CA	36.61	−121.89
San Francisco, CA	37.81	−122.47
Bolinas Lagoon, CA	37.91	−122.68
Point Reyes, CA	38.00	−122.97
Arena Cove, CA	38.92	−123.71
North Spit, CA	40.77	−124.22
Crescent City, CA	41.75	−124.19
Port Orford, OR	42.74	−124.50
Charleston, OR	43.35	−124.32
South Beach, OR	44.63	−124.05
Garibaldi, OR	45.56	−123.92
Cape Disappointment, WA	46.30	−124.00
Toke Point, WA	46.71	−123.97
Westpoint, WA	46.90	−124.11
La Push, WA	47.91	−124.64
Neah Bay, WA	48.37	−124.60

#### Mean monthly sea level (MMSL)

MMSL variability is due to a multitude of processes including seasonal variability, large-scale climate variability, such as ENSO, and local surface temperatures that make deterministic numerical modeling of local monthly anomalies difficult. Instead, a time series of MMSL anomalies were approximated via a stochastic climate emulator^40^ that used a multiple linear regression model to link MMSL (the Predictand) to the PCs of monthly mean SST and SLP anomalies (the Predictors). This model was used to fill gaps in tide gage observation time series and populate MMSL values for time periods before the establishment of a gauge or after its decommissioning.

First, the observed MMSL at a tide gauge was calculated as the monthly mean recorded water level minus the local sea-level rise trend, accomplished by detrending the available water level time series, and a three-year moving water level average. This process removed decadal-scale trends and variability from the observed MMSL time series.

Next the model predictors were defined. The first predictor was the three dominant PCs of the monthly mean SST anomaly time series for the period of 1979 to 2016 extracted from the Extended Reconstructed Sea Surface Temperature Version 4^41^ dataset for a rectangular region from 120° E to 280°E and 5°N to 5°S at a resolution of 2.5°. Along the West Coast, the first SST anomaly PC generally reflects ENSO and the resultant water level changes during that event. The other two most dominant modes reflect regional and basin-wide seasonal anomalies. The three dominant SST anomaly PCs captured much of the SST variability (67%) across the time period.

The second predictor were the PCs of the mean monthly SLP anomalies. Local weather phenomena can be represented by SLP fields, which capture high- and low-pressure systems, and their squared gradients (SLPG), which relates to wind stress over the ocean. Regional SLP and SLPG timeseries were extracted from NOAA’s Climate Forecast System Reanalysis^42^ for period of 1979 to 2016 in a 400 km grid with a resolution of 0.5° around the region of the tide gauge. First, the daily values were extracted and the monthly means at each grid node were calculated. The PCs of these parameters were generated and the number of PCs utilized was variable, but had the requirement that combined they represent at least 98% of the observed variance in the SLP and SLPG patterns.

#### Storm surge (SS)

SS was simulated at each tide gauge by tying the PCs of the regional maximum daily SLP fields around each location to SS observations. First, a timeseries of SS measurements was calculated from each tide gauge record. From the tide gauge water level, the astronomical tide and SLR trend were subtracted from the water level record. Next a 3-year moving average was subtracted to remove long-term water level trends, and finally the calculated MMSL timeseries described above was removed. This process yielded the hourly water level variations that could be attributed to local pressure systems. From this time series, the daily maximum water levels were extracted, representing SS.

Next the regional SLP fields and SLPGs were extracted from the Climate Forecast System Reanalysis using the same technique and extent as for the MMSL. In this case, the daily maximum value was extracted at each grid node as opposed to the monthly mean. Again, the PCs of SLP and SLPG were calculated, with the number of PCs utilized needing to represent at least 98% of the observed variance in the SLP and SLPG patterns. The PCs were used as predictors to estimate local SS (the predictand) using a distance-weighted K-Nearest Neighbors algorithm regression (see Anderson and others^40^ for further detail). The model was trained using 10 study cases that each divided the observed SS time series into 10 subgroups. In any one study, 9 of the subgroups were used for calibration of the model (where data were available from the tide gauge) and 1 subgroup was validated and the reconstruction of SS was extracted (including time steps not provided by the tide gauge). The validation subgroups were not coincident, so by the end of the 10 studies, 10 different subgroup time periods were validated, extracted, and collated into one continuous reconstruction of daily SS maxima.

#### Modeling wave run-up (*R*_*2%*_*)*

The last component of Eq. 1 is the *R*_*2%*_ term. *R*_*2%*_ is the 2% exceedance level of vertical uprush above the SWL due to wave action. Unlike the previous water level data, wave *R*_*2%*_ is a highly localized process that is controlled by nearshore wave transformations and cross-shore morphologies. To ultimately generate an hourly record of TWLs at each transect, it was first necessary to obtain hourly wave data at each transect that was then used to calculate *R*_*2%*_.

Sea level variations induced by wave breaking require an accurate definition of the wave climate at nearshore depths. Hourly nearshore wave data were simulated in a multi-step process. First, sixty-one years (1948–2008) of validated, long-term, hourly hindcast deep-water wave data were extracted from the GOW database^11^ at 7 deep-water locations offshore of the West Coast (Table 3). Second, the offshore wave conditions were distilled into 500 combinations of representative sea-states (wave heights, wave periods, and wave directions) best representing the variability of data time series at the GOW output points following the methodology of Camus and others^43^. These sea states were used as boundary conditions and propagated to the nearshore using the two-dimensional Simulating Waves Nearshore (SWAN) numerical spectral wave model^44–46^, which simulates nearshore wave transformations by solving the spectral action balance equation. The inputs used by SWAN are significant wave heights (*H*_*s*_), peak wave periods (*T*_*p*_), and wave directions at a rectilinear boundary, in this case as direct output from the 500 GOW wave sea states. It also incorporates gridded bathymetries that influence propagation and standard assumptions of wave breaking and multi-wave interactions to accurately transition deep-water waves into nearshore, shallow water waves that can be used for empirical run-up and overtopping assessments. The nearshore results of the SWAN model were used to recreate hourly wave data at the nearshore using a transfer function^43^. Five hundred combinations were selected as this amount was determined to be able to accurately interpolate a 61-year timeseries of nearshore conditions while saving on the total number of downscaling wave simulations. Thus, this approach is computationally less expensive than a traditional lookup table where many more combinations of wave parameters need to be modeled to capture all of the potential variability.

**Table 3:** Names and locations of GOW output locations as inputs for nearshore downscaling via SWAN models.

GOW Output Designation	Latitude (°N)	Longitude (°E)
NAWC33	34.08	−121.98
NAWC32	36.69	−123.98
46214	37.95	−123.47
NAWC31	39.28	−125.56
NAWC30	42.20	−126.16
NAWC29	45.23	−126.01
NAWC28	47.86	−126.93

Waves were first simulated in coarse, rectilinear, regional grids and downscaled into smaller, finer-resolution nested grids. The alongshore resolution of the fine grids were approximately 100 m. For California, bathymetry and SWAN grid configurations were adapted from Erikson and others^47^. The finer resolution California grids were curvilinear to optimize the run-time. One exception to this configuration was in Southern California, where a medium sized grid was nested within the coarse grid to better resolve the effects on wave propagation between the Channel Islands. The finer grids were then nested within this rectilinear medium grid. Washington and Oregon wave model grids were entirely rectilinear, with 3 sets of grid sizes. The Oregon grids were developed by García-Medina and others^48^ and Allan and others^49–52^. The Washington grids were generated from NOAA Centers for Environmental Information coastal digital elevation models (DEM) (https://www.ngdc.noaa.gov/mgg/coastal/coastal.html) bathymetry data. These grids were subsequently processed into a 3-tiered, nested set up. The first is a coarse regional grid at 900 m, with a smaller 300 m grid nested within this regional grid, and finally a nearshore grid of 100 m for each nearshore location. Most default SWAN settings were used; however, 24 frequency and 72 directional bins were utilized to adequately simulate wave refraction in the finest grids. For coarser grids in Southern California, 34 frequency bins were necessary to adequately resolve wave propagation through the Channel Islands. Additionally, the frequency range was set to be 0.0418 to 1 Hz to better capture high-energy events^53^. The 500 shallow water wave conditions were extracted from the finest SWAN grids at each cross shore transect at the 15 m isobath. The conditions at each transect were then reconstructed into an hourly time series of *H*_*s*_ and *T*_*p*_ for 1948–2008 using multidimensional interpolation approaches^43^.

#### Extracting nearshore wave conditions

Wave conditions were extracted at the 15 m isobath and the wave heights were converted to deep water conditions using linear theory. In some cases, the transects never intersected bathymetry contours as deep as 15 m, such as along the flanks of a headland, and wave conditions were extracted at the deepest depth along that transect as far out as 3 km from the coast. This is a limitation of keeping the transect shore-normal at crenulated shorelines causing the transects to orient away from deep, open water. In the cases where wave heights were extracted from the SWAN models at a depth less than 10 m, these conditions were unmodified.

#### Run-up (*R*_*2%*_) calculations by shoreline type

**Fig. 4:**
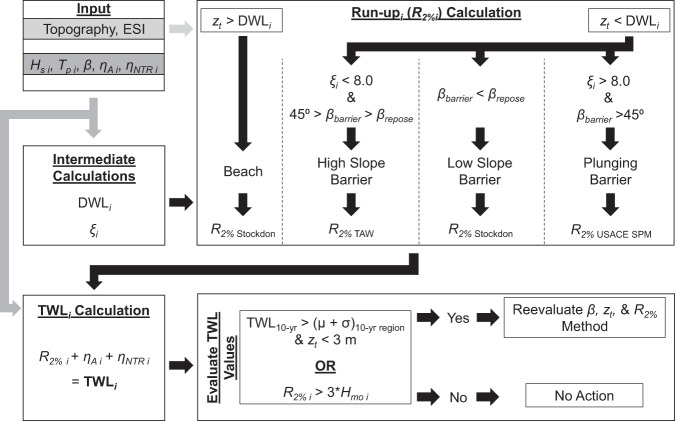
Flow chart detailing total water level (TWL) calculations from wave model output, Environmental Sensitivity Index (ESI) category, and Digital Elevation Model (DEM) topography including run-up methodology selection and TWL magnitude evaluation. The runup method selected is indicated by the *R*_*2%*_ subscript and TWL_10-yr_ refers to the 10-year return period TWL at the transect. (µ + σ)_10-yr region_ refers to the average 10-year TWL event for a predefined region including the transect plus the standard deviation of those regional values. The subscript *i* indicates the values used in TWL calculation at an individual time step.

Due to the variation in shoreline type along the West Coast, *R*_2*%*_ was calculated using a combination of three different empirical formulae, each calibrated for a different shoreline type: run-up along (1) sandy beaches^36^, (2) retaining structures^54^, and (3) vertical walls^55^. The application of *R*_2*%*_ methods is summarized in Fig. 4 and described below.

*R*_*2%*_ on sandy beaches was computed using the Stockdon and others^36^ parameterization:

2 $${R}_{2{\rm{ \% }}}=1.1\left(0.35\,\tan \,\beta {\left({H}_{O}{L}_{O}\right)}^{\frac{1}{2}}+\frac{{\left[{H}_{O}{L}_{O}\left(0.563\tan {\beta }^{2}+0.004\right)\right]}^{\frac{1}{2}}}{2}\right)$$

where *β* is the beach slope in radians, *H*_*o*_ is the incident deep-water wave height, and *L*_*o*_ is the incident deep-water wavelength. Where the coastal profile was primarily a sandy shoreline, possibly including dunes, or the backshore slope was generally <36° (the angle of repose for sand) and the other run-up approaches listed in Fig. 1 were not appropriate for the environment, the Eq. 2 formulation was used. In practice, steep conditions were rarely used in Eq. 2 and often other *R*_*2%*_ methods described below were found to be more appropriate.

The second *R*_*2%*_ formula was the Technical Advisory Committee for Water Retaining Structures (TAW) formula^54^ for use when the DWL exceeded *z*_*t*_ and 36° < *β*_*b*_ < 45°, where *β*_*b*_ is the composite slope of the barrier (described as *β*_*barrier*_ in Fig. 4). DWL is the combination of the still water level, wave setup, and infragravity wave motions Eq. 3.

3 $$DWL=SWL+1.1\left[\left(0.35\,\ast \,\beta \,\ast \,\sqrt{\left({H}_{s}\,\ast \,{L}_{o}\right)}\right)+\frac{\left(0.06\,\ast \,\sqrt{\left({H}_{s}\ast {L}_{o}\right)}\right)}{2}\right]$$

where SWL is the still water level, *β* is the foreshore or beach slope, *H*_*s*_ is the wave height extracted at the 15 m bathymetric contour, and *L*_*o*_ is the deepwater equivalent wavelength. The first term within the brackets is an approximation of wave setup and the second is the infragravity swash^36^. The DWL is an important component contribution to the calculated *R*_*2%*_ and TWL as it incorporates infragravity motions and set up caused by waves that the incident wave swash acts upon. In effect, TWL differs from DWL as the TWL incorporates incident swash (wave run-up, *R*_*2%*_) on top of the DWL.

For this study, TAW (Eqs. 4 & 5) was adapted for use in non-dike environments following the FEMA guidelines for the Pacific Coast flooding analysis^56^ and Allan and others^49^. The TAW *R*_*2%*_ formulation is given as:

4 $$\begin{array}{l}{R}_{2{\rm{ \% }}}={H}_{mo}\left(1.75{\gamma }_{b}{\gamma }_{f}{\gamma }_{\beta }{\xi }_{m-1.0}\right)\\ {\rm{for}}\;0 < {\gamma }_{b}{\xi }_{m-1.0} < 1.8\end{array}$$

and

5 $$\begin{array}{l}{R}_{2{\rm{ \% }}}={H}_{mo}\left(1.0{\gamma }_{f}{\gamma }_{\beta }\left(4.3-\frac{1.6}{\sqrt{{\xi }_{m-1.0}}}\right)\right)\\ {\rm{for}}\;{\gamma }_{b}{\xi }_{m-1.0} > 1.8\end{array}$$

where γ_*b*_ is the influence factor for a berm (if present and was only calculated along exposed wave-cut platform profiles), γ_*f*_ is the influence factor for slope roughness, γ_*β*_ is the influence factor for oblique wave attack (not addressed in this effort), and *ξ*_*m–1.0*_ is the breaker parameter defined as:

6 $${\xi }_{m-1.0}=\frac{\tan \,{\beta }_{b}}{{\left(\frac{{H}_{mo}}{{L}_{m-1.0}}\right)}^{0.5}}$$

where tan*β*_*b*_ is the slope of the barrier, *H*_*mo*_ is the spectral significant wave height at the toe of the barrier, *L*_*m–1.0*_ is the deepwater wavelength: $\left(\frac{g{T}_{m-1.0}^{2}}{2\pi }\right)$, where *T*_*m-1.0*_ is calculated as *T*_*p*_/1.1. *H*_*mo*_ is calculated as (DWL- *z*_*t*_) * 0.78. Wave direction influences were not considered in this study as wave data were extracted at nearshore and were assumed to have a shore-normal incidence angle. To reconstruct the wave directions like *H*_*s*_ or *T*_*p*_, the angular directions must be deconstructed into Cartesian x and y vectors, reconstructed into hourly data independently, and then combined into polar coordinates. While the reconstruction methodology saves time by reducing the total number of SWAN runs necessary, it is still time intensive. Therefore, each *R*_*2%*_ calculation considers a shore-normal incidence to save time and still provide a conservative estimate of *R*_*2%*_ and its effects.

An approximate reduction factor was applied for structure and substrate material where appropriate. Along exposed rock, concrete, or cliff environments, this reduction factor was set to 1.0 (no reduction). However, in the case of revetments and loose material, the reduction factor was set to 0.65 for boulder rubble (ESI 6D), 0.55 for riprap (ESI 6B and 8 C), and 0.7 for gravel beaches (ESI 6A) per NHC^56^ and Allan and others^49^. The reduction influence of a berm was considered along exposed rocky platform (ESI 2 A) shorelines. The exposed platform was determined to simulate a concrete berm along a dike. There may have been other profiles where this reduction may be appropriate, but these profiles would need to be identified manually. Given the total number of profiles, these locations were unable to be reasonably identified beyond the preliminary categorization of the ESI dataset. The calculation for this and other reduction factors can be found in the FEMA West Coast guidelines^56^. Engineered berms fronting seawalls and dikes were not resolved within the DEM and were not included. For further explanation of TAW (Eqs. 4 & 5) and its application along natural coastlines, see van der Meer^54^ and Allan and others^49^.

A composite slope was calculated to represent *β*_*b*_ over the elevation range of the wave setup plus the SWL to the TWL calculated using *R*_*2%*_ computed via Eq. 2 for the incident wave condition. A composite slope accounts for a fronting beach and the cliff/engineered structure and the range of potential slopes between rather than just the slope of the cliff/engineered structure. Where the TWL exceeded *z*_*c*_, *z*_*c*_ was instead used to define the upper bounds of the composite slope. Along plunging profiles, often no toe could be identified due to the LiDAR data not extending below the water line. In these cases, the cliff face was computationally extended following the cliff face slope to the elevation of MSL and that point was marked as *z*_*t*_ of the cliff. In these instances, the lower bound used to estimate the composite slope was defined using interpolated foreshore beach slope (*β*) used to calculate DWLs.

In instances where the calculated *R*_*2%*_ via TAW was unrealistic, determined by producing extremely large *R*_*2%*_ values (for example, 30 m) for the incident wave conditions, the composite slope was redefined using an iterative approach^57^ whereby an initial estimate of *β*_*b*_ was calculated as the slope from SWL–1.5*H_*mo*_ to SWL+1.5*H_*mo*_ along the transect and initial *R*_*2%*_ estimate was calculated using this preliminary slope using Eqs. 4 and 5). A final *β*_*b*_ was then calculated as SWL–1.5*H_*mo*_ to the *R*_*2%*_ estimate level and a final run-up calculated using this slope.

There are a number of limitations for the TAW (Eqs. 4 and 5) methodology as it was applied within this study and required careful consideration in its application. The TAW methodology is reliant on the breaker parameter, *ξ*_*m–1.0*_ (Eq. 6), to calculate relative *R*_*2%*_ (Eqs. 4 and 5). However, TAW (Eqs. 4 and 5) is only valid for *ξ*_*m–1.0*_ values of 1.8 to 8–10, and the methodology is only meant to be utilized for *β*_*b*_ < 45°^57^. In this study, large *ξ*_*m–1.0*_ values were calculated when *z*_*t*_ was low in elevation (below 3 m NAVD88), regardless of which method was used to calculate the composite *β*_*b*_, often in plunging cliff environments and where *z*_*t*_ of engineered structures such as riprap extended below the observed water line. In these cases, the computed/approximated *β*_*b*_ values were generally steep or *H*_*s*_ at *z*_*t*_ was large, resulting in *ξ*_*m–1.0*_ > 8. A greater *ξ*_*m–1.0*_ produced unrealistically large *R*_*2%*_ magnitudes (again on the order of 20–30 m in some cases), and the *R*_*2%*_ methodology had to be modified (Fig. 4).

A final iteration in the *R*_*2%*_ methodology after an initial calculation with TAW was subsequently considered to correct errant *R*_*2%*_ estimations. This final process was dictated by a series of operational ranges for the composite *β*_*b*_ and are described in the Fig. 4 flow chart. Where *β*_*b*_ was < 36°, the toe elevation low (<3 m), and *R*_*2%*_ values deemed unrealistically low (by producing a final *R*_*2%*_ elevation lower than the calculated DWL) or unrealistically high (large *R*_*2%*_ magnitudes regardless of *β*_*b*_ calculation method), *R*_*2%*_ was recalculated via Eq. 2 regardless of substrate using *β*. It was determined that Eq. 2, produced a more realistic *R*_*2%*_ value in this scenario. If *β*_*b*_ was > 45° or the *R*_*2%*_ magnitudes for 36° < *β*_*b*_ < 45° were still unrealistically large due to large *ξ*_*m–1.0*_ magnitudes, it was assumed that the conditions were highly reflective. In these instances, the formulation for *R*_*2%*_ along a vertical wall was used to adapt to the extremely reflective conditions. The relation for *R*_*2%*_ along a vertical wall is defined in the U.S. Army Corps of Engineers Shoreline Protection Manual (SPM)^55^ as:

7 $${R}_{2{\rm{ \% }}}=1.5\times {H}_{mo}$$

In these cases, the slope of the cliff or engineered structure was generally not vertical. However, it was determined that this approximation more accurately represented *R*_*2%*_ magnitudes in highly reflective conditions versus TAW (Eqs. 4 and 5), and there is not a more reliable empirical approximation for *R*_*2%*_ along steep cliffs and bluffs or for large *ξ*_*m–1.0*_ in FEMA or U.S. Army Corps of Engineers documentation.

In summary, if a sandy beach backed by a cliff/structure/barrier, the Stockton *et al*.^36^ was first used in every time step. However, if the dynamic water level^36^ (DWL, defined in Eq. 3) exceeded *z*_*t*_ of a barrier, run-up was we recalculated for that time step using the TAW formula (Eqs. 4 & 5). If the barrier *z*_*t*_ was not inundated by the DWL, only Eq. 2 was used for the *R*_*2%*_ calculation. However, if the DWL exceeded *z*_*t*_ and *β*_*b*_ was > 45° or *ξ*_*m–1.0*_ > 8, such as along a plunging cliff, Eq. 7 was used. In all other cases, Eq. 2 was used.

#### Total water level (TWL) and dynamic water level (DWL) return period calculations

**Fig. 5:**
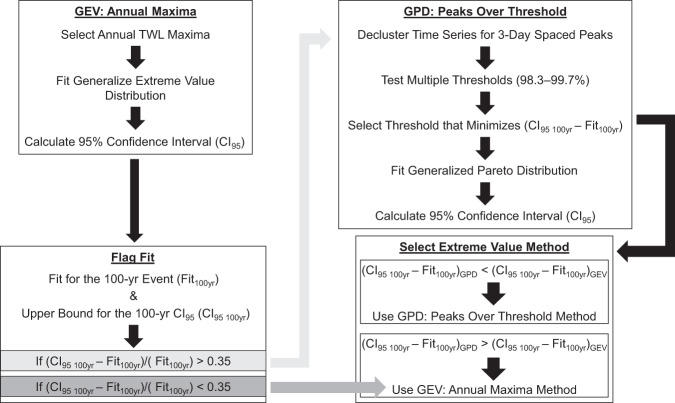
Flow chart detailing selection of extreme value analysis method to generate the extreme total water level (TWL) and return periods. The shaded arrows in grey indicate the next step in the process if the conditions for the Confidence Interval in the corresponding boxes are met. The dark grey arrow indicates that the annual maxima GEV method is selected without testing the peaks over threshold method.

TWLs at each transect were calculated by a linear superposition approach following Serafin and others^37,38^. The estimation of MSL, local *η*_*A*_ levels at the shoreline, and interpolated *η*_*NTR*_ values were summed for each time step to determine the SWL. The wave *R*_*2%*_ was added to the SWL to compute the time series of TWLs. Return-period TWLs and DWLs (extreme water level magnitudes associated with return period events) were derived via two extreme value analyses fits utilizing the hourly TWL and DWL estimates: annual block maxima fit to a generalized extreme value (GEV) distribution and a peaks-over-threshold approach fit to a generalized pareto distribution (GPD). The process for selecting an extreme value method at an individual profile is summarized in Fig. 5.

Annual maximum TWLs were extracted from the hourly TWL time series (from 1948 to 2008) at each transect. The annual maxima of TWLs were then fit to the GEV distribution^58^:

8 $$G\left(z,\mu ,\sigma ,\xi \right)=exp\left\{-{\left[1+\xi \left(\frac{z-\mu }{\sigma }\right)\right]}^{-1/\xi }\right\}$$

$${\rm{defined}}\;{\rm{on}}\;\left\{z:1+\frac{\xi (z-\mu )}{\sigma } > 0\right\},$$

where – ∞ < *μ < *∞, *σ* > 0, and – ∞ < *ξ < *∞ and *μ* is the location parameter, *σ* is the scale parameter, and *ξ* is the shape parameter. These parameters are estimated by maximizing the log-likelihood function^58^. This approach was used to define the TWLs associated with the 1‐, 2‐, 5‐, 10‐, 20‐, 25‐, 50‐, 100‐, 250‐, and 500-year return periods. The annual maxima were determined to be the greatest value for the October to March months each year; these months correspond with the greatest wave energy in the Pacific Ocean during the boreal winter months^59,60^. This approach has limitations^49^ as extreme data are inherently discarded when selecting annual maxima. However, the long time period of 61 years, provides a sufficient number of data points to fit to a GEV distribution. It was determined through testing that the annual maxima method represented extremes of the North Pacific and resultant West Coast TWLs well. In testing, multiple events/year tended to yield poorer fits due to changes in *R*_*2%*_ methodology and resultant TWL along complex morphologies. Exceeding a threshold (such as the DWL exceeding the toe of a cliff) and transferring from one *R*_*2%*_ method [such as Eq. 2] to another [such as Eqs. 4 and 5] along a single transect changes the dynamic between the incident wave conditions and the resultant *R*_*2%*_. The rate of *R*_*2%*_ increase with increasing wave magnitudes is different for these methods. Additionally, as *β*_*b*_ is calculated dynamically for each time step, it tends to become greater for larger *H*_*mo*_, increasing *R*_*2%*_ estimations. In practice, along some transects, the non-linear changes in these relationships resulted in jumps within of the relative magnitudes between methods of the largest values, creating conditions for poor fits. Given the scope of the study and its use of automation, the careful consideration required at each time step at transect to abate this issue could not be adequately addressed. At most profiles, more values produced similar if not the same results as the annual block maxima.

Estimates of the return level for a particular return period year with probability *p* of occurrence using an annual block maxima GEV analysis^58^:

9 $${\rm{Return}}\;{\rm{Period}}\;{\rm{Year}}=\left\{\begin{array}{c}\mu -\frac{\sigma }{\xi }\left[1-{\left\{-\log \left(1-p\right)\right\}}^{-\xi }\right],{\rm{for}}\;\xi \ne 0,\\ \mu -\sigma \log \left\{-\log \left(1-p\right)\right\},{\rm{for}}\;\xi =0,\end{array}\right.$$

Confidence intervals were obtained using the delta method^58^, which assumes normality of the maximum likelihood estimate of a scaler function derived from the data. 95% confidence intervals (CI_95_) of the return levels were generated to help evaluate goodness-of-fit.

When the data fit to Eq. 8 was poor, defined here as (CI_95 100yr_ – Fit_100yr_)/(Fit_100yr_) > 0.35 (threshold determined by testing), the data were declustered to yield maximum values at least 3 days apart and instead fit to a GPD function with the extremes being selected using a Peaks-Over-Threshold method^58^. Here, CI_95 100yr_ is the upper bound for the 100-yr 95% confidence interval and Fit_100yr_ is the modeled return value for the 100-yr event. A decreasing threshold (from 99.7 to 98.3%) was used to produce threshold magnitudes from which the one that minimized the CI_95 100yr_ to Fit_100yr_ ratio was selected while still producing a subjectively good fit of the data to the GPD distribution. The GPD is given as^58^:

10 $$H\left(y,\sigma ,\xi \right)=1-{\left(1+\frac{\xi y}{\widehat{\sigma }}\right)}^{-1/\xi }$$

where *y* denotes threshold excesses, *ξ* is the GEV shape parameter, and $\widehat{\sigma }$ is the scale parameter related to the GEV parameters by $\widehat{\sigma }$ = *σ* + *ξ* (*u* – *μ*). For a GPD-Poisson analysis, the N-year return level (*y*_*N*_) can be obtained as:

11 $${y}_{N}=\mu +\frac{\sigma }{\xi }\left[{\left(N{n}_{y}{\zeta }_{u}\right)}^{\xi }-1\right]$$

where *n*_*y*_ is the number of observations per year; *N* is the return period in years; and *ζ*_*u*_ is the probability of an observation exceeding the threshold *u*. When the CI_95 100yr_ to Fit_100yr_ ratio produced by Eq. 10 was smaller than the CI_95 100yr_ from the GEV fit in Eq. 8, the GPD methodology was used to determine the return values. However, if the GPD CI_95 100yr_ to Fit_100yr_ ratio was larger, only Eq. 8 was used.

**Fig. 6:**
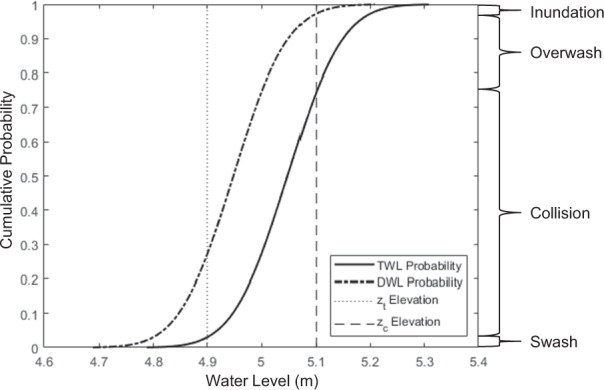
Example cumulative density function describing probabilities of potential total water levels (TWLs) and dynamic water levels (DWLs) plotted against *z*_*t*_ and *z*_*c*_. The impact regime and fraction of the cumulative probability function is indicated on the right. The bold, black line represents the TWL probability; the bold, dash-dot line corresponds to the DWL probability; the dotted line is *z*_*t*_; and the dashed line is *z*_*c*_. Swash and collision probabilities are solely defined by the TWL probability cumulative density function (*P*CDF) intersecting *z*_*t*_ and *z*_*c*_. Overtopping probability is defined by the difference of both TWL and DWL *P*CDF curves exceeding the *z*_*c*_, and inundation probability is solely defined by the DWL *P*CDF exceeding *z*_*c*_.

Once the best fit model was selected for each transect, the return levels were computed to define a normal probability distribution around each return period value. In practice, this process was defined in the same way as the confidence intervals used in determine the best fit. A 95% confidence interval is associated with a specific probability of occurrence (*p* = 0.95). This probability is used to determine a Z score that helps define the confidence interval buffer around the projected mean using the delta method^58^. Therefore, a normal distribution of values for each return period can be numerically populated by evaluating the value of these buffers at a range of *p* = 0 to 1. After defining a normal probability distribution for a return period event, these were transformed into cumulative density functions (CDFs) of probability for each return level at a transect (Fig. 6).

#### Adjustments for unrealistic extreme total water levels (TWLs)

Despite the efforts of the processes above, some of resultant return period TWLs were found to be unrealistically large, as defined below. A final sweep of these errant transects indicated that the affected profiles almost always were selected for use with TAW (Eqs. 4 & 5); therefore, transects that used TAW and produced unrealistic values were flagged and reprocessed. In almost all cases, the affected profiles were along rocky or cliff environments with a small fronting beach such that the *z*_*t*_ was extremely low in elevation and with a steep *β* estimate. A low *z*_*t*_ and large slope results in an overprediction of *H*_*mo*_ at the *z*_*t*_ location, which increases the estimation of *ξ*_*m–1.0*_ such that *ξ*_*m–1.0*_ > 8. Large *ξ*_*m–1.0*_ values often exceeded the applicable range for TAW (Eqs. 4 & 5), causing *R*_*2%*_ errors that were only detectible after the initial calculation.

Erroneous transects were flagged by determining if a transect TWL exceeded a qualitative threshold based of the calculated return TWL evets within a region. After defining the 10-yr TWL event at each transect from the extreme value analysis, transects where the local 10-yr TWL event exceeded a regional mean + regional standard deviation for all 10-yr events calculated using TAW (Eqs. 4 & 5) and had a *z*_*t*_ < 3 m (Fig. 4) were flagged as unrealistically large. The definition of the regional mean and standard deviation was assigned by convenience; in this case, the project data were divided by alongshore county, and the regional means and standard deviations were calculated at that varying county-by-county scale, despite an understanding that the political boundaries do not follow morphology. Ultimately, this variability in the averages was deemed appropriate as a checking mechanism because it limited comparison between wholly different regions (such as a study-area wide mean for all of Washington, Oregon, and California), was conveniently calculated from previous data output formats, and within a smaller radius of one county to the next, these means did not shift substantially but did so over the entire study area (i.e., the regional means for southern California were appropriately different than for northern California).

The threshold of *z*_*t*_ < 3 m contribution to the unrealistically large TWLs was determined through evaluating these conditions at a number of profiles with varying morphologies. In Allan and others^49^, the profiles for which TAW (Eqs. 4 & 5) was adapted generally had *z*_*t*_ > 3 m, and comparably shallower *β* as the beaches were often wider than many of the rocky coastlines within this study. However, with *z*_*t*_ > 3 m, water level overprediction with TAW (Eqs. 4 & 5) lessened as *β* became smaller and *H*_*mo*_ values were more constrained to smaller values by the higher *z*_*t*_ elevation. Additionally, *R*_*2%*_ magnitudes greater than 3*H_*mo*_ for a time step were flagged as potentially unrealistic because the *H*_*mo*_ calculation could lead to large wave height estimates in locations with low *z*_*t*_ and a lack of offshore information to guide wave transformations and wave setup in the breaker zone. Future characterizations of nearshore wave climate could help address this limitation.

Once transects with errant 10-yr TWL estimations were identified, several steps were considered to improve the estimates of the TWL time series and return period magnitudes. First, the morphological conditions and *R*_*2%*_ methodology were reassessed and the TWL time series was recalculated (Fig. 4). *z*_*t*_ and *z*_*c*_ were reexamined to determine if points further on shore were more appropriate to describe the cliff/barrier feature. In this process, *z*_*t*_ and *z*_*c*_ were redefined as well as *β*. The *β* used for this correction was not averaged with the surrounding locations as there was the possibility that the influence of the surrounding transects could increase the revised *β* estimate and yield erroneous *R*_*2%*_ values.

Next, the *R*_*2%*_ method was altered such that all cliff/barriers whose maximum slope was < 36° utilized Eq. 2. All flagged instances where *ξ*_*m–1.0*_ > 8 used Eq. 7. Although not vertical, these large *ξ*_*m–1.0*_ magnitudes indicated reflective conditions more appropriately served by Eq. 7. Finally, any values in the calculated *R*_*2%*_ time series greater than 3*H_*mo*_ were initially replaced with *R*_*2%*_ calculated via Eq. 2. If the resultant *R*_*2%*_ values were still greater than 3*H_*mo*_, Eq. 7 was used for those time steps with the assumption that they were still unrealistically large. This approach utilizes *R*_*2%*_ methods that are not intended for a rocky or lower slope environment, but produced more realistic *R*_*2%*_ values than TAW in many cases. However, these values were less accurate than other profiles whose morphology more easily lent itself to the appropriate TAW formulation. It is recommended that future work should identify empirical equations that can adequately assess *R*_*2%*_ along barriers with low *z*_*t*_ values and little offshore depth information. Once these conditions were evaluated, the TWL time series and return level events were recalculated for the affected transects.

### Calculation of storm impact

Return-period extreme event TWL probabilities were compared to the onshore topography at each transect to determine the probability of that event causing coastal change (e.g., erosion and flooding). For this analysis, the calculated return period TWLs and DWLs with their associated probability distributions were utilized. To estimate the probability of collision, overwash, and inundation (pCOI, proxy estimates for coastal change) at each transect, these water level distributions were compared to critical elevations of the extracted morphology (*z*_*t*_ and *z*_*c*_) along each transect elevation profile^2^ (Fig. 6).

Four storm-impact regimes are defined within pCOI to provide a framework for examining the likelihood of coastal change for any given event^20^. The four regimes are swash (TWL < *z*_*t*_), collision (*z*_*t*_ <  = TWL < *z*_*c*_), overwash (TWL > = *z*_*c*_), and inundation (DWL > *z*_*c*_). In the swash regime, hydrodynamic forces are seaward of the toe, resulting in little to no morphological change or flooding. The collision regime indicates the potential for dune face/bluff erosion or structural damage once the toe is surpassed. With an overwash regime, the water level is above the crest, generating potential landward sediment transport and mild flooding. Finally, inundation results in the backshore being completely exposed to hydrodynamic forces^20^. Inundation is predicted if the DWL exceeds *z*_*c*_, then it is assumed that much of the transect is below the local water level for an extended period of time.

*z*_*t*_ or *z*_*c*_ at each transect was compared to the probability (*P*) CDF for each return period event. For example, in Fig. 6, the probability of the TWL = 4.9 m (*z*_*t*_ elevation) and TWL = 5.1 m (*z*_*c*_ elevation) is identified on the TWL *P*CDF. If the elevation of *z*_*t*_ intersects the TWL *P*CDF the associated *P* at that location is recorded as *P*_swash_ (in this example *P*_swash_ = 0.03). *P*_Collision_ calculated by finding *P* at the intersection of the TWL *P*CDF curve and *z*_*c*_ and then subtracting *P*_swash_ (*P*_Collision_ = 0.72). Finally, *P*_overwash_ and *P*_inundation_ were calculated by finding the intersection of the TWL *P*CDF and DWL *P*CDF with *z*_*c*_ (Fig. 6). If inundation occurs, so must overwash; therefore, *P*_overwash_ was further modified as *P*_overwash_final_ = *P*_overwash_ – *P*_inundation_. Ultimately, in Fig. 6, *P*_overwash_final_ = 0.21 and *P*_inundation_ = 0.04. These probabilities add up to 1 to represent the full range of possible regime outcomes. In the instances where water levels do not exceed the *z*_*t*_, the *P*_swash_ = 1 and the rest of the regimes are zero, and if the water levels do not exceed the crest, *P*_overwash_final_ = 0 and *P*_inundation_ = 0.

Finally, a days-per-year analysis for each transect determined how many representative days TWLs could result in each impact regime^38^. For this, daily maxima TWL and DWLs were extracted from the 61 year-long hourly time series at each location and then each daily maximum was categorized into an impact regime based on *z*_*t*_ and *z*_*c*_ using the above methodology. The days-per-year occurrence for a generic year at a profile was calculated as:

12 $$DP{Y}_{regime}=\frac{Day{s}_{regime}}{Day{s}_{total}}\times 365.25$$

where *DPY*_*regime*_ is the days-per-year that the profile experiences the regime in question (for example, 40 days of overwash), *Days*_*regime*_ is the total number of days in the daily maxima time series that the profile experiences a specific regime, and *Days*_*total*_ is the total number of days within the daily time series.

## Data Records

The results from this report are available for download as comma-separated value (csv) ASCII files at (10.5066/P95FBGZ1)^61^. Each file contains information for a single return period at >25,000 locations spaced approximately 100 m apart along the open West Coast MHW line from the Mexican Border to the Strait of Juan de Fuca. There are a total of 10 separate files to coincide with the total number of modeled return periods (1-, 2-, 5-, 10−, 20-, 25-, 50-, 100-, 250-, and 500-year events). The rows of the ASCII files correspond to a single transect. Each column corresponds to individual parameters for that transect. These parameters are: transect end latitudes and longitudes, modeled return period TWL, modeled return period DWL, transect *z*_*t*_, transect *z*_*c*_, MHW, storm impact scale regime, and a days-per-year analysis of storm impact scale regime over 1948–2008. Each elevation value (TWL, DWL, *z*_*c*_, *z*_*t*_, and MHW) is paired with the latitude and longitude of where that elevation intersects the transect elevation profile.

## Technical Validation

### Non-tidal residuals

MMSL (predictand) was modeled by assuming a linear relationship between MMSL and the predictors (PCs of SST, SLP, and SLPG). Please see Anderson and others^40^ for greater detail concerning the climate emulator technique and derivation of the multivariate regression model. The resultant linear model for each location was calibrated and validated against the MMSL timeseries derived from the corresponding NOAA tide gage. Overall, the linear model models represented the MMSLA well for each gauge, with the greatest root-mean square error (RMSE) being 0.16 mm (Table 4). The modeled SS time series were validated against tide gauge SS measurements. In general, the modeled data fit the observed data less well than for the MMSL but was still acceptable. The maximum calculated RMSE did not exceed 0.05 m (Table 4), giving confidence that despite variation in scatter, the approximation for SS was adequate.

**Table 4:** Root mean squared error of modeled versus observed MMSL and SS time series at NOAA tide gauge stations.

Station Location	RMSE	
	MMSL (mm)	SS (m)
San Diego, CA	0.16	0.05
Monterey, CA	0.15	—
San Francisco, CA	—	0.03
North Spit, CA	0.11	—
Port Orford, OR	—	0.03
South Beach, OR	0.09	—
Toke Point, WA	0.09	0.04

Overall, MMSL was represented well by the modeled data, showing no significant bias and fitting the observed time series well.

### Wave propagation approach

**Fig. 7:**
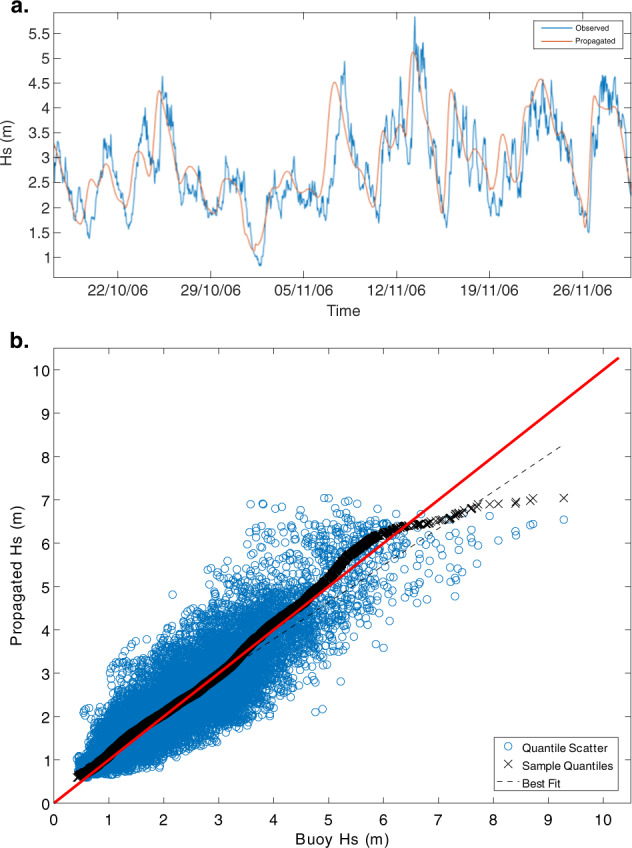
Significant wave height (*H*_*s*_) propagation versus observed conditions for NDBC station 46027 Northwest of Crescent City, Calif. (**a**) Observed (blue line) versus propagated (orange line) *H*_*s*_ time series. (**b**) Quantile-Quantile plot of observed and modeled reconstruction *H*_*s*_ values for 27641 matching reconstructed and buoy records between 2005 and 2009. The red line represents the 1:1 line indicating perfect fit, the blue circles represent the quantile scatter, the black Xs represent a sample quantile pairing at increasing thresholds, and the dashed black line represents the best fit linear regression line for the quantile scatter.

Wave downscaling accuracy was evaluated at locations within the SWAN grid domains and GOW output locations coincident with or neighboring National Data Buoy Center (NDBC) buoys (https://www.ndbc.noaa.gov/). These simulations were evaluated by three methods. The first was to calculate the RMSE of the modeled versus observed time series of wave parameters (Fig. 7a). The second was to calculate the index of agreement between the observed versus propagated time series^62^. These validation statistics are summarized in Table 5. Third, the wave heights at each location were semi-quantitatively assessed via quantile-quantile plots (Fig. 7b).

**Table 5:** Root mean squared error and index of agreement validation statistics of hourly wave buoy time series of *H*_*s*_ and *T*_*p*_ versus modeled propagations.

Station ID	Latitude (°N)	Longitude (°E)	*H*_*s*_		*T*_*p*_	
			RMSE (m)	Index of Agreement	RMSE (s)	Index of Agreement
46047	32.403	−119.536	0.14	0.99	1.70	0.91
46086	32.491	−118.035	0.33	0.92	1.21	0.95
46069	33.670	−120.200	0.09	1.00	1.61	0.91
46025	33.749	−119.053	0.25	0.94	2.24	0.87
CDIP 141	34.100	−119.167	0.05	0.99	1.58	0.88
46053	34.252	−119.853	0.16	0.98	3.00	0.79
46054	34.265	−120.477	0.17	0.99	2.10	0.87
CDIP 131	34.356	−119.476	0.09	0.98	1.31	0.92
46011	34.956	−121.019	0.21	0.99	1.90	0.89
46028	35.741	−121.884	0.05	1.00	1.63	0.92
46042	36.789	−122.404	0.16	0.99	1.30	0.94
46012	37.363	−122.881	0.39	0.96	1.53	0.92
46026	37.755	−122.839	0.06	1.00	2.59	0.81
46013	38.238	−123.307	0.35	0.96	2.67	0.80
46014	39.235	−123.974	0.11	1.00	1.55	0.92
46022	40.720	−124.531	0.17	0.99	1.76	0.89
46027	41.852	−124.382	0.11	1.00	1.85	0.89
46015	42.779	−124.874	0.18	0.99	1.80	0.89
46050	44.677	−124.515	0.09	1.00	1.99	0.86
46089	45.925	−125.771	0.24	0.99	1.58	0.92
46029	46.143	−124.485	0.20	0.99	1.98	0.86
46041	47.353	−124.742	0.26	0.99	1.84	0.88
46087	48.493	−124.726	0.31	0.98	1.74	0.88

Buoy stations are from the National Data Buoy Center unless otherwise noted.

In general, the wave models adequately captured wave transformations at deeper locations, generally indicating high indices of agreement and relatively low RMSE, as listed in Table 5. This is important to first validate the GOW output and the SWAN model setup before any bathymetry-driven wave transformations. Additionally, along large stretches of the northern West Coast, shallow water buoys are sparsely positioned, so deep-water buoy data were in many cases all that were available for comparison within the GOW time period. These agreements along with the fit of the quantile-quantile plots to the 1:1 line improved when only comparing swell conditions (*T*_*p*_ > 8 s). Certain locations closer to shore, such as NDBC 46027, still had good agreement between observed and modeled wave conditions, but the quantile-quantile plots indicated that the largest observed values were not captured by the GOW wave model and subsequent reconstruction (Fig. 7b).

Discrepancies were due to two factors. First the GOW dataset, while output at hourly intervals, is driven by 3-h winds^11^, and was simulated at a resolution of 1.5° longitude and 1° latitude which reduces variability of the wave conditions that can be represented at model output points (Fig. 7a), leading to lower *H*_*s*_ values compared to the observed data and slight temporal offsets of peaks and troughs between the time series. Additionally, there is a consistent time-lag of a few hours in the observed wave parameters versus the modeled due to wave travel times to the nearshore not being simulated. Overall, the GOW captures average deep ocean wave data well^11^. However, downscaling and extracting those conditions at exact output locations cannot simulate other local processes that lead to increased variability. Second, the model boundaries were far offshore to be coincident with GOW output locations; therefore, any local effects such as nearshore winds and storms that were not captured by the coarse resolution, deep water GOW dataset were not represented. This is exemplified along northern locations, such as NDBC station 46027 located offshore to the northwest of Crescent City, Calif., where the occasional alongshore coastal gale and other localized storms decrease the agreement in the extreme analyses (Fig. 7b). This pattern is representative for most of the northern (Northern California through Northern Washington) locations. However, the majority of the extremes are well represented in the downscaled SWAN models, emphasizing that most extreme conditions and TWLs are primarily a result of swell-wave forcing from far afield. The TWLs provided by this effort should be viewed as driven by swell conditions, which is representative of the large wave conditions in most transects the majority of the time.

### Uncertainties, limitations, and assumptions

To produce extreme TWL estimations and pCOI values at a high resolution across the West Coast a number simplifying assumptions were made and therefore sources of uncertainty need to be considered when utilizing these results. First, the LiDAR dataset had a vegetated vertical accuracy of ±0.204 m and non-vegetated vertical accuracy of ±0.116 m. These accuracies likely did not significantly affect the results as vegetation along the coastal profiles is often absent or sparse, especially in the southern half of the study and extreme water level variations were on the order of meters

Much of the input information was drawn from predefined NOAA models and datasets. Water level datum estimates were established by NOAA’s VDatum tool, which has an average maximum cumulative uncertainty of 9.8 cm for the California Coast, 18.3 cm for Oregon, and 15.4 cm for Washington (https://vdatum.noaa.gov/docs/est_uncertainties.html). These errors could minutely affect the placement of the MHW shoreline along each transect elevation profile and the calculation of TWLs incorporating MSL estimates. The NOAA ESI database is continuously updated but some locations have not been reevaluated within the last 10–15 years. Some of these locations may have since been modified but are not accounted for within this study. The accuracy of the ESI dataset was validated manually in many locations and was found to be accurate in most. The study region is too large to adequately validate each transect location’s ESI value, so the methodology to determine *R*_*2%*_ methods and relevant morphology may have inaccuracies at limited locations.

The bathymetry datasets utilized in this study were aggregated to include the most recent and highest resolution available at the time of this study. Many of the selected bathymetries had different collection years and resolutions, which could introduce unrealistic output in the wave model results. In areas lacking new, high-resolution bathymetry, older, coarser datasets were used to supplement the recent data. For downscaling wave propagation into the nearshore, it was necessary to assume that the bathymetries had not changed dramatically since collection. Regionally, it is unlikely that an older bathymetric dataset would dramatically affect wave model results. However, at the highly local scale, it is very possible that bathymetries have changed significantly and would affect TWL values. Therefore, it is not recommended that the modeled results be applied to a small-scale location absent of a specific assessment.

*η*_*A*_ information was modeled at an ~1 km alongshore interval with the assumption that spatial variations along the open coast at this resolution were likely minimal. *η*_*NTR*_ values were solely modeled at NOAA tide gauges and linearly interpolated between these stations. Local variations brought about by for example pocket beaches surrounded by cliff headlands or stretches of open coast where SS differs compared to adjacent tide gauges within bays and harbors are likely the greatest source of error in the SWL. The sparse observational network of storm surge measurements limits detailed and robust evaluation of this uncertainty, but because storm surges rarely exceed 0.5 m along this narrow-shelved coastline, the errors introduced are small relative to the other TWL components. MMSL variations are more widely distributed along the West Coast and are better captured by this approach. Therefore, results should be viewed as approximating a SS regime for a location between tide gauges with the goal of providing realistic TWL estimates, but highly localized storm effects are lost.

Wave downscaling utilized inputs from the GOW model, which provides hourly wave conditions driven by three-hour winds offshore. The three hourly winds can reduce the temporal variability of wave conditions compared to observational datasets, and additionally, locally generated wave energy across the narrow continental shelf was not explicitly simulated. Whereas locally generated wave energy contributes to nearshore wave energy, it is well known that open coast impacts along the West Coast are largely driven by remotely generated swell and regionally generated seas^9,63–65^. Additionally, the wave downscaling created a small temporal offset between the observed and modeled wave conditions as the propagation time from the model boundary to the observation platform was not accounted for. Despite this offset, RMSE values calculated most NDBC buoys were generally small. This offset may result in slight temporal mis-alignments between water level and wave signals, but this effect is expected to be ameliorated as the data are aggregated over 61 years, thus representing a large set of possible and realistic conditions.

The *R*_*2%*_ methodology utilizes empirical equations that have ideal use conditions. Given the morphological diversity of the West Coast, these equations had to be applied for non-ideal conditions, especially relating to highly reflective environments like cliffs. This limitation was necessary as the selection of empirical relations within the literature is limited for use along steep coastal slopes, such as cliff shorelines and cliff-backed beaches. The empirical relations were therefore selected based on wide applicability, previous usage along cliff and beach environments, and ability to handle reflective conditions. Future work should utilize numerical modeling or updated empirical relations to better ascertain the potential *R*_*2%*_ along these transects.

The extreme value analyses were computed and verified programmatically. There were too many transects to manually validate each extreme value fit and select the most appropriate threshold/method for each transect. Therefore, the calculated return period TWLs may not reflect accurate values for all locations, but rather are a best attempt given the West Coast-wide scale. Additionally, some return period TWLs may be erroneously large or small (such as less than the transect’s MHW value) but were not caught by programmatic quality control efforts. A simple outlier analysis was conducted to replace these erroneous water level and probability data with a not a number (NaN) designation in the final dataset provided with this report. For each transect, the mean and standard deviation of the TWLs for all transects within 0.01° were calculated. If the transect TWL exceeded the regional TWL mean by more than three times the standard deviation, or if it was less than its associated MHW value, it was replaced with NaN. This process was utilized for each return period. These erroneous transects were few in number compared to the total number of transects (<1.5%). Finally, the probability distribution for each return period TWL were assumed to be normally distributed but may not in fact be so depending on the local conditions.

This effort represents the aggregation of a multitude of variables from many sources to project extreme water levels. The impact of these assumptions is likely small, and no quantification of these uncertainties is provided with the final data products. Future work should seek to incorporate nearshore coastal storms and their localized SS as well as improved *R*_*2%*_ methodology, such as bespoke equations for the West Coast environments or numerical *R*_*2%*_ modeling. The pCOI analysis and return period TWLs should therefore be viewed as guiding approximations and the best estimates available for the large study area.

## Data Availability

All data processing and analysis of existing datasets and the generation of TWLs, DWLs, return periods, and probabilistic extreme water level impacts were carried out by a custom MATLAB code library developed specifically for this project. A repository of these codes and templates can be found at https://github.com/Climate-Shope/West_Coast_TWLs.git for access and download. Due to the complexity of the scripts and backing data, please contact Li Erikson at lerikson@usgs.gov for assistance in implementation if necessary.
